# Allelopathy as a Strategy for Biological Invasion: *Calotropis procera* and Its Impact on Plant Succession

**DOI:** 10.1002/cbdv.202501711

**Published:** 2025-11-29

**Authors:** Bruno Melo de Alcântara, Paulo Henrique Calixto Santana, Felipe Rufino dos Santos, José Weverton Almeida‐Bezerra, José Galberto Martins da Costa, Delmira da Costa Silva, Cláudia Maria Furlan, Maria Arlene Pessoa da Silva

**Affiliations:** ^1^ Universidade Regional do Cariri Crato Brazil; ^2^ Department of Chemical Biology Universidade Regional do Cariri Crato Brazil; ^3^ Department of Biological Sciences Universidade Estadual de Santa Cruz Ilheis Brazil; ^4^ Department of Botany Institute of Biosciences University of São Paulo Brazil

**Keywords:** allelopathy, biological invasion, exotic species, semiarid

## Abstract

This article reports on the allelopathic potential of *Calotropis procera* (Aiton) W.T. Aiton foliage on *Handroanthus impetiginosus* (Mart. ex DC.) Mattos. Predominant species in semiarid environments, especially in the Caatinga of the Northeast, where it acts as an invasive species. The leaves of *C. procera* were mixed with soil from the Caatinga and left to decompose for 90 days. The control group consisted only of soil from the Caatinga, Northeast, Brazil. The germination test was conducted in quadruplicate (30 seeds/treatment). For growth, 30 replicates per treatment were used, each with one seedling of the recipient species. The identification of the chemical constituents of *C. procera* was assessed by GC–MS. The data was submitted to the one‐way ANOVA test. Residues from the decomposition of *C. procera* litter negatively affected seedling germination, with a reduction of up to 80% in the treatments evaluated. The phytochemical analysis showed the presence of 18 compounds, grouped into six main classes. Among the main compounds identified were: α‐amyrin (C_30_H_50_O) with 2.69% and O‐acetyl‐β‐amyrin (C_32_H_52_O_2_) with 6.19% concentration. Significant variation was observed in the thickness of the tissues and regions of the radicle, hypocotyl and leaves of *H. impetiginosus*. *C. procera* litter has a negative allelopathic potential on the recipient species, which could lead to their reduction in the natural environment.

## Graphical abstract

Stages of the study on the allelopathic potential of Calotropis procera (Aiton) W. T. Aiton. The image shows the collection of plant material, the decomposition of organic matter, the identification of bioactive compounds, and the effects on the germination of Handroanthus impetiginosus (Mart. ex DC.) Mattos.

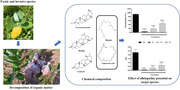

## 1 Introduction

Ecological functioning, defined as the flow of energy and materials between the abiotic and biotic components of an ecosystem, is often compromised when exotic and invasive species are introduced into natural environments [1]. These species cause ecological and socioeconomic impacts, affecting native biodiversity and agricultural production systems [2, 3]. The main factors contributing to the introduction and spread of invasive species include globalization, the growth of international trade and tourism, climate change (e.g., global warming), and alterations in biogeochemical and water cycles due to agricultural activities [4]. Invasive exotic species display certain characteristics that allow them to occupy and establish themselves in other natural environments, such as fast growth, high seed production and dispersal rates, high germination rate, absence of natural predators, long periods of flowering and fruiting, and allelopathic potential [1, 5]. The production of secondary metabolites with the capacity to alter the germination patterns and development of other species has been identified as one of the mechanisms of the biological invasion process [6].

Among plant species with recognized invasive potential in natural environments and agricultural areas, *Calotropis procera* (Aiton) W.T. Aiton (Apocynaceae), a plant native to Asia and Africa, has been reported as a weed in countries with areas of arid climate such as Egypt, the Arabian Peninsula, Pakistan, Afghanistan, India, and Brazil [7, 8, 9, 10, 11, 12]. In Brazil, this species is popularly known as “flor‐de‐seda,” “leiteira,” “ciumeira,” and/or “algodão‐de‐ceda” [13, 14]. *C. procera* was introduced into Brazil at the beginning of the 20th century, in the city of Recife, state of Pernambuco, later spreading throughout the northeast region and other states in Brazilian territory [15, 16, 17]. This species is considered difficult to control because its easy propagation and dispersal, combined with its high germination rate and large number of seeds, favor its occupation and biological invasion in natural environments. [17]. This species has been reported in diverse studies about the botanical composition of exotic vegetation introduced in different regions of the globe, such as Australia, Fuerteventura (Canary Islands), desert regions of Rajasthan, India, and Madagascar [18, 19, 20, 21, 22].

The species is usually recorded in open fields, roads, deserts, agricultural and grassland areas, as well as natural environments, causing impacts on local flora and fauna [19, 20, 21]. Several studies reported the degree of biological invasion by *C. procera* in Caatinga areas of the northeast region of Brazil [23, 24, 25, 26]. According to Fabricante [25], *C. procera* had an average of 11.9 individuals per sample unit (8.3 adults and 3.5 regenerating individuals), demonstrating its significant presence and abundance in the Caatinga area studied. This species has a relevant degree of phytotoxicity and has caused toxic incidents involving grazing animals and humans [27, 28]; The phytotoxic potential of *C. procera* is related to the diversity of bioactive substances present in its composition, including saponins, phenolic compounds, triterpenes, norditerpene esters, flavonoids, proceresterol, steroidal hydroxyketone, stigmasterol, β‐sitosterol, and procerain [29]. Due to its invasive and chemical features, the allelopathic potential of *C. procera* has been evaluated, as many of the compounds found in this species display this property [30].

Floristic composition studies have demonstrated the occurrence of *C. procera* in different areas of Caatinga in the northeast region of Brazil [31, 32]. Among the native species present in these areas is *Handroanthus impetiginosus* (Mart. ex DC.) Mattos, a species popularly named “ipê‐roxo,” “ipê‐rosa,” and “ipê‐preto,” with occurrence re‐ported in South America, Central America, and Mexico [33]. *H. impetiginosus* has pharmacological and economic applications and is commonly used in reforestation and urban afforestation programs [34]. Recent studies have emphasized the need for conservation of this species, mainly in Caatinga areas, due to its intense use by the civil and timber industry, and also losses by deforestation. It is classified as near‐threatened (NT) species in the Red List of Brazilian Flora [35, 36].

The species *C. procera* has a great capacity to tolerate a wide variation of edaphoclimatic conditions, producing biomass throughout the year, even under extreme environments as found in the Brazilian semiarid region [37, 38]. As a consequence, there is a considerable accumulation of biomass and decaying organic matter in the soil. During the decomposition process, there is a release of secondary metabolites into the environment, which can negatively affect surrounding species. In this context, this study aimed to evaluate the allelopathic potential of *C. procera* leaf decomposition on the germination and morphoanatomy of *H. impetiginosus* seedlings, highlighting how the release of secondary metabolites can influence ecological interactions and the regeneration dynamics of native species in the Caatinga.

## 2 Results and Discussion

### 2.1 Analysis of Physicochemical Parameters

The physicochemical analysis of the Caatinga soil demonstrated an increase in most of the micro‐ and macronutrients as a result of adding *C. procera* litter. However, the levels of aluminum (Al), hydrogen (H), and aluminum saturation (M), were reduced. Aluminum was reduced to 0 Cmolc/dm^3^ in all treatments compared to the control group (CK) (Table 1). The reduction in these aluminum availability values, possibly due to the action of compounds released during leaf decomposition (such as organic acids), could reduce stress, allowing for better germination and growth rates of plant species. In ecological terms, the results suggest that *C. procera* leaves do not affect other plants solely through direct toxins, but also alter soil chemistry, potentially modulating the local plant community.

**TABLE 1:** Physicochemical characteristics of Caatinga soil in the control and in treatments with different concentrations of *Calotropis procera* litter.

Treatments	P (mg/dm^3^)	pH (H_2_O)	Ca (Cmolc/dm^3^)	Mg (Cmolc/dm^3^)	Na (Cmolc/dm^3^)	K (Cmolc/dm^3^)	Al (Cmolc/dm^3^)	H (Cmolc/dm^3^)	S (Cmolc/dm^3^)	CEC (Cmolc/dm^3^)	V (%)	M (%)
CK	3	4.50	0.50	0.50	0.01	0.04	0.75	1.06	1.1	2.9	37	42
T1	14	5.90	1.65	0.60	0.02	0.31	0.00	0.74	2.6	2.6	78	0
T2	36	6.70	2.20	0.70	0.06	0.70	0.00	0.16	3.7	3.7	96	0
T3	56	7.00	2.20	1.00	0.04	0.50	0.00	0.16	3.7	3.7	96	0

*Note*: CK: Control; T1: 9 g/kg; T2: 18 g/kg; T3: 27 g/kg; pH: hydrogen potential; P: phosphorus; K: potassium; Na: sodium; H: hydrogen; Al: aluminum; Ca: calcium; Mg: magnesium; S: sum of bases; CEC: cation exchange capacity; V: base saturation capacity; M: aluminum saturation.

In terrestrial ecosystems, the majority of the addition of macro‐ and micronutrients to the soil occurs in response to the decomposition of the litter of existing species, among which are nitrogen (N), phosphorus (P), and potassium (K) [39]. This could explain the increase in some essential nutrients observed in the different treatments with the addition of *C. procera* litter (Table 1) to the Caatinga soil. The process of decomposition of organic matter results in the availability of nutrients (through mineralization), meeting the energy and nutrient demands of decomposing organisms and plants [40].

The soil pH increased in all treatments compared to the control (CK), ranging from 5.9 to 7.0, whereas the control had a pH of approximately 4.50. One of the effects of adding organic matter to the soil is the neutralization of acidity due to the release of bicarbonate ions, reducing the solubility of aluminum and its availability in the environment [41]. The correlation between aluminum values and the increase in pH observed in this study can be confirmed when compared to other studies, where the decomposition of organic matter resulted in a decrease in aluminum values and an increase in average pH values at different concentrations [42, 43]. In the work by Silva [42], the influence of *Azadirachta indica* litter on the physicochemical parameters of the substrate showed an increase in pH values in the different treatments, ranging from 6.60 to 7.70. In addition, a decrease in the concentration of aluminum in the substrate to 0 Cmolc/dm^3^ was noted in all treatments, simi‐lar to the data observed in the present study.

According to Roy [44], high levels of acidity or alkalinity can alter the germination parameters of seedlings. However, no considerable variation was observed that could influence the germination of the species under study, so this factor was disregarded in our work. Regarding the control group (CK), the more acidic pH (4.50) did not affect the germination of the receptor species, showing a germination rate above 90%. The variation in the increase of pH values among the treatments (T1, T2, and T3), with pH values classified as slightly acidic to neutral, could not have caused negative effects on germination, since the ideal pH for the germination of most forest species is between 6.0 and 7.5, aligning with the results observed in Table 1 for osmotic potential [45].

The soils of the Caatinga tend to exhibit low chemical activity, with a predominance of acidic soils, low values for the sum of bases (S), and low cation exchange capacity (CEC), resulting in lower retention of available nutrients, and low base saturation (V%) [46]. These results were observed in Table 1, where the soil of the control group (CK) showed base saturation below 50%, indicating it is a dystrophic soil (V% < 60%) with a low nutrient concentration. Regarding the treatments, from the first concentration of *C. procera* litter (T1), a 41% increase in base saturation was already observed. In the last two concentrations (T2 and T3), values were close to 100%, indicating that the organic matter made the soil eutrophic (V% > 60%).

Among the effects associated with the increase of organic matter in the soil, we can mention the increase in base sum (S) values and CEC, in response to the increase in basic cations such as calcium (Ca), magnesium (Mg), and potassium (K) [47]. In addition, it can lead to a reduction in soil acidity due to the complexation of aluminum, and the reduction of hydrogen ions (H^+^), as a result of the transformation of organic acids into CO_2_ and water (H_2_O) [48, 49].

### 2.2 Chromatographic Analysis of the Extract by GC–MS

**FIGURE 1:**
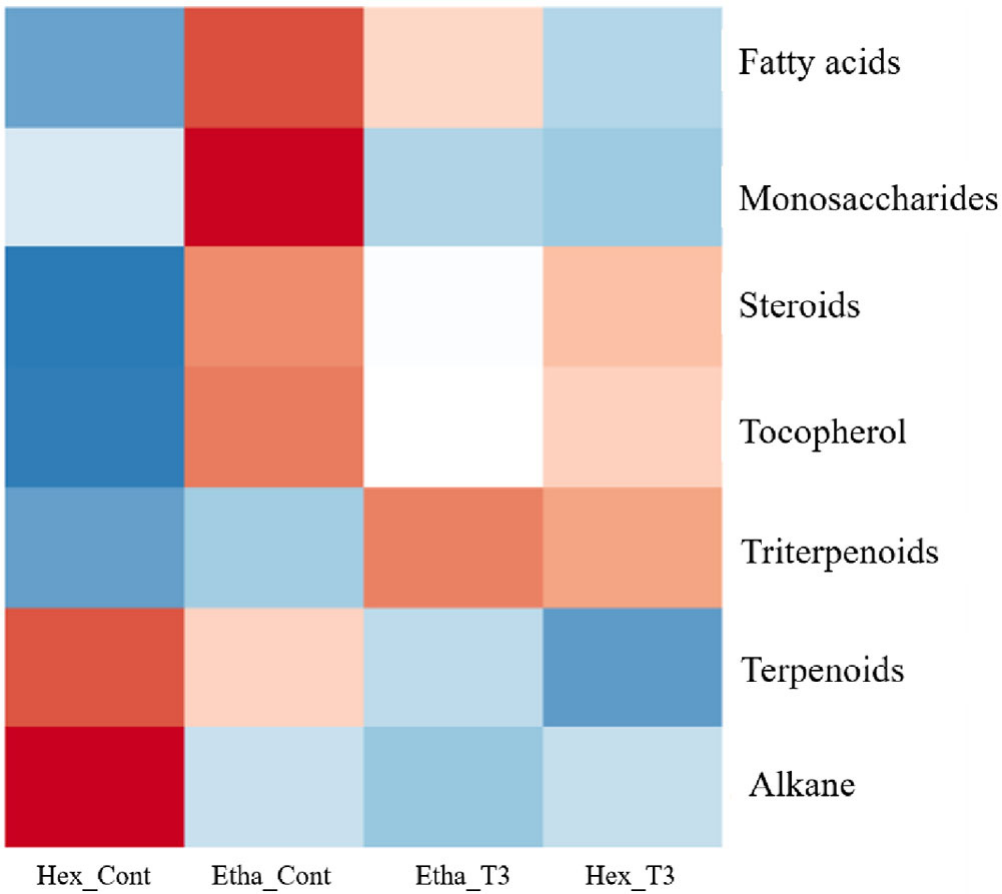
Correlation of the metadata of the main classes of metabolites detected in the experiment. Heatmap showing similarities and patterns for hexane and ethanolic extracts of the control group (CK), and in the Caatinga soil after 90 days of *Calotropis procera* leaf litter decomposition at a concentration of 27 g/kg (T3). The highest and lowest abundance values are colored in red and blue, respectively.

The GC–MS analysis revealed the presence of 18 compounds, grouped into six main classes: alkanes, fatty acids, terpenes (triterpenes and diterpenes), tocopherols, disaccharides, and phytosterols. Among the compounds identified in the soil containing *C. procera* litter, 12 were observed in both extracts (hexane and ethanol), five were found only in the ethanol extract, and one in the hexane extract (Tables 2 and 3). Six fatty acids, five triterpenoids, two tocopherols, two alkanes, one diterpenoid, one disaccharide (sugar), and one steroid were annotated. For the hexane extract, triterpenoids (13.63%), phytosterols (1.12%), and fatty acids (0.38%) were the main classes of compounds detected in *C. procera* (Figure 1). Regarding the ethanol extract, triterpenoids also represented the major class (12.64%), followed by phytosterols (1.87%) and fatty acids (1.77%) (Figure 1).

**TABLE 2:** Compounds identified (GC/MS) in the Caatinga soil after 90 days of leaf litter decomposition of *Calotropis procera* (ethanolic extract).

Compounds	Retention time (min)	%	Molecular formula	Molecular mass(g/mol)	Properties	References
Fatty acids						
Pentadecanoic acid	26.118	0.05	C_17_H_34_O_2_	270.5	Not reported	—
Linoleic acid, ethyl ester	30.524	0.35	C_20_H_36_O_2_	308.5	Hypocholesterolemic;nematicide;antiarthitic;insectifuge; antieczemic and allelopathic potential	[60, 61, 62, 63]
9‐Octadecenoic acid	30.732	0.12	C_20_H_38_O_2_	310.5	Anticancer and antimicrobial	[64, 65]
Oleic acid	31.618	0.8	C_18_H_34_O_2_	282.5	Antitoxidant and allelopathic potential	[66]
Methyl arachidate	33.444	0.06	C_21_H_42_O_2_	326.6	Not reported	—
Lignoceric acid	40.997	0.39	C_24_H_48_O_2_	368.6	Not reported	—
Alkene						
1‐Tricosene	32.545	0.12	C_23_H_46_	322.6	Not reported	—
Disaccharides						
Maltose	39.616	0.61	C_12_H_22_O_11_	342.30	Not reported	—
Tocopherol						
γ‐Tocopherol	43.280	0.39	C_28_H_48_O_2_	416.7	Not reported	—
α‐Tocopherol	44.915	0.45	C_29_H_50_O_2_	430.7	Not reported	—
Phytosteroids						
Campesterol	46.538	1.87	C_28_H_48_O	400.7	Anti‐inflammatory and immunomodulatory properties, allelopathic potential	[67]
Terpenoids						
Phytol	30.857	0.11	C_20_H_40_O	296.5	Anticancer and anti‐inflammatory and allelopathic potential	[68, 69, 70]
Triterpenoids						
α‐Amyrin	47.768	2.69	C_30_H_50_O	426.7	Anticancer	[71]
Lupeol	48.341	1.02	C_30_H_50_O	426.7	Anticancer	[72]
O‐acetyl‐β‐amyrin	48.890	6.19	C_32_H_52_O_2_	468.8	Not reported	—
Ursolic acid	50.994	1.24	C_30_H_48_O_3_	456.7	Anti‐inflammatory and antioxidant	[73]
Betulin	52.124	1.5	C_30_H_50_O_2_	442.7	Antifungal and antimicrobial activity	[74]

**TABLE 3:** Compounds identified (GC/MS) in the Caatinga soil after 90 days of leaf litter decomposition of *Calotropis procera* (hexane extract).

Compounds	Retention time (min)	%	Molecular formula	Molecular mass (g/mol)	Properties	References
Fatty acids						
Linoleic acid, ethyl ester	30.513	0.07	C_20_H_36_O_2_	308.5	Hypocholesterolemic; nematicide; antiarthitic; insectifuge; antieczemic and allelopathic potential	[60, 61, 62, 63]
Oleic acid	31.617	0.1	C_18_H_34_O_2_	282.5	Antitoxidant and allelopathic potential	[66]
Lignoceric acid	40.993	0.21	C_24_H_48_O_2_	368.6	Not reported	—
Alkene						
*n*‐Heptatriacontane	51.431	0.6	C_37_H_76_	521.0	Not reported	—
Disaccharides						
Maltose	39.606	0.15	C_12_H_22_O_1_	342.30	Not reported	—
Tocopherol						
γ‐Tocopherol	43.275	0.09	C_28_H_48_O_2_	416.7	Not reported	—
α‐Tocopherol	44.900	0.25	C_29_H_50_O_2_	430.7	Not reported	—
Phytosteroids						
Campesterol	46.518	1.12	C_28_H_48_O	400.7	Anti‐inflammatory and potential allelopathic immunomodulatory properties	[67]
Triterpenoids						
α‐Amyrin	47.729	2.35	C_30_H_50_O	426.7	Anti‐inflammatory	[71]
Lupeol	48.324	1.82	C_30_H_50_O	426.7	Antimicrobial and anticancer	[72]
O‐acetyl‐β‐amyrin	48.853	6.16	C_32_H_52_O_2_	468.8	Not reported	—
Ursolic acid	50.992	1.2	C_30_H_48_O_3_	456.7	Antimicrobial and anticancer	[73]
Betulin	52.112	2.1	C_30_H_50_O_2_	442.7	Antifungal and antimicrobial activity	[74]

Among the volatile compounds released during the decomposition of organic matter are mainly those from the terpene group, which are subsequently stored in the soil and can affect surrounding species [50]. This group of bioactive compounds is known for its allelopathic activity associated with the germination and growth of plant species [51]. In the study by Naser [52], chromatographic analysis of the crude leaf extract of *C. procera* was conducted using GC–MS, and it predominantly showed compounds within the classes of terpenes, fatty acids, and steroids, similar to the findings of this study. Al‐Rowaily [53] and Rehman [54] also observed a higher composition of terpenoids in the oil and crude leaf extract of *C. procera*, respectively.

Regarding the comparative analysis of the main classes of compounds observed in the treatment T3 and the control group (CK), it was observed that the control showed a higher composition of compounds from the classes of fatty acids, monosaccharides, steroids, tocopherols, and terpenes, varying according to the type of solvent used during the extraction. For treatment T3, a greater abundance was observed in three classes: steroids, tocopherols, and triterpenoids (Figure 1). The chemical analysis of the soil from the control group (CK) showed a variation in abundance according to the type of solvent used in the extraction (ethanol and hexane), with alkanes being more abundant in the hexane extract and monosaccharides in the ethanol extract. Since the soil used in the experiment did not undergo sterilization, to maintain the natural microbiota and composition of the natural soil, it was expected to observe the presence of compounds in the chemical analysis for the control group, due to the natural release of litter by the species from the original environment, and the natural biota of the soil (Figure 1).

Soils in natural environments generally display a rich biodiversity of microorganisms present on the surface and within the soil substrate [55]. This vast composition of microorganisms (fungi, bacteria, and protozoa) reflects the complex diversity of interactions and the richness of soil microbiota [56]. The presence of these microorganisms plays essential roles in the ecosystem, including influencing the chemical composition and structure of the soil [57]. Therefore, it is crucial to understand the response of allelochemicals released by the donor species in association with the soil biota, taking into account all the processes involved in the allelopathic potential.

The chromatographic analysis of the soil containing *C. procera* litter showed a predominant presence of compounds from the triterpene class in both solvents, with greater evidence in the ethanol extract. The hexane extract exhibited a greater variety of chemical composition, while the ethanol extract showed significant abundance mainly for fatty acids and triterpenoids (Figure 1).

**FIGURE 2:**
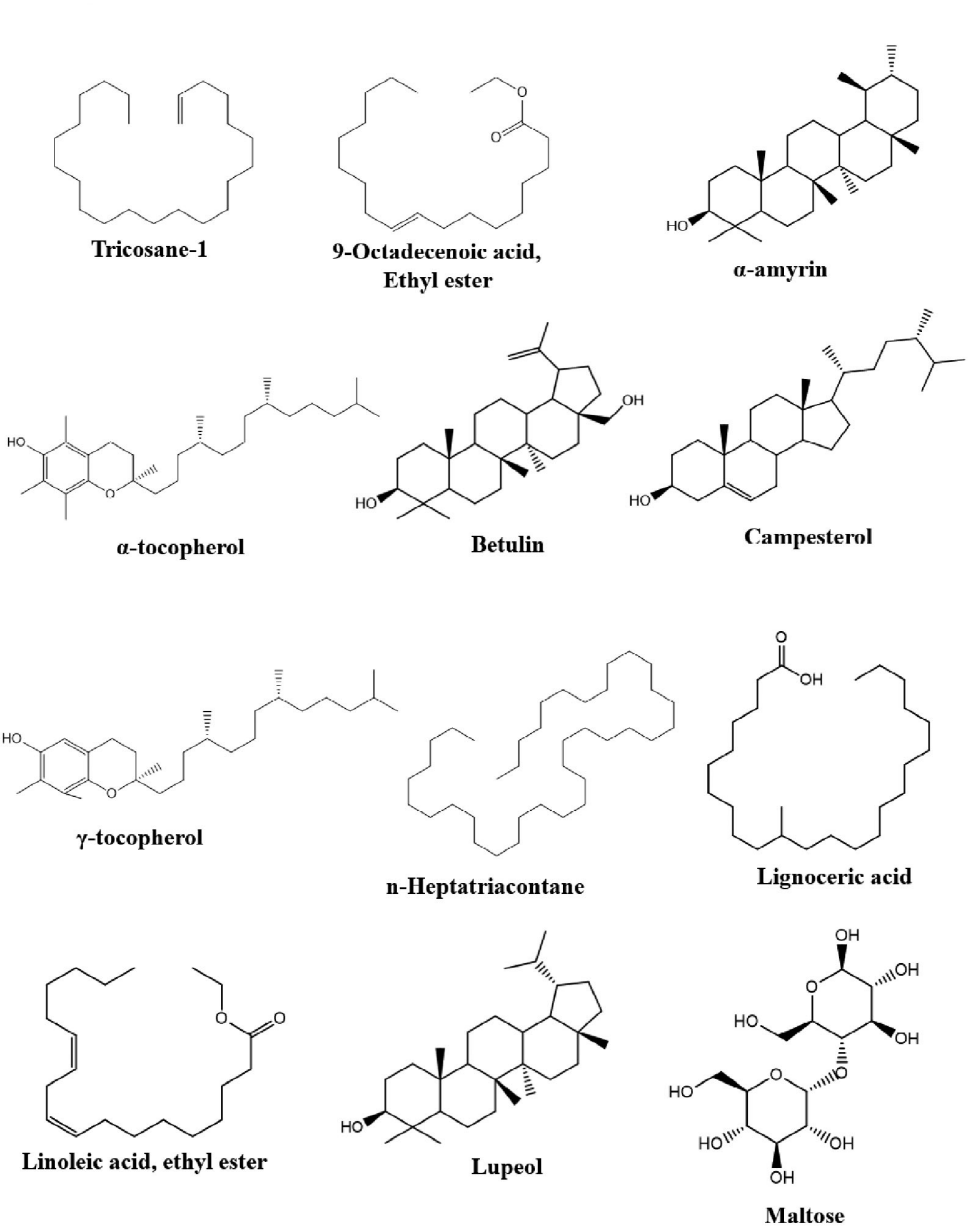
The structures of the specific compounds found after the foliar decomposition of *Calotropis procera* for both extracts used in the extraction process (ethanol and hexane).

Among the major compounds observed in the Caatinga soil containing the leaf litter of *C. procera* are: O‐acetyl‐β‐amyrin (C_32_H_52_O_2_), α‐amyrin (C_30_H_50_O), and betulin (C_30_H_50_O_2_), which are compounds within the class of terpenes (triterpenoids) (Figure 2). Naser [65] also found the presence of the following compounds, which are common to this study: octadecanoic acid (C_20_H_38_O_2_) and α‐tocopherol (C_29_H_50_O_2_) (Figure 2). Genetic and environmental factors are crucial in determining the chemical composition of a species, and variations can occur among individuals of the same taxonomic group [58]. Biotic factors (pathogens, competition, and predators) and abiotic factors (light, water, soil, and available nutrients) directly influence the biosynthesis and fluctuation of plant secondary metabolites [59].

In the study by Al‐Rowaily [53], the chemical composition of the essential oil from the aerial parts of *C. procera* was evaluated in two distinct regions, Saudi Arabia and Egypt, to determine if environmental and climatic factors that could influence the chemical composition of this species. Variation in the chemical composition was observed between the two locations. However, in both locations, the presence of the compound phytol (C_20_H_40_O) was noted.

### 2.3 Germination Test

**FIGURE 3:**
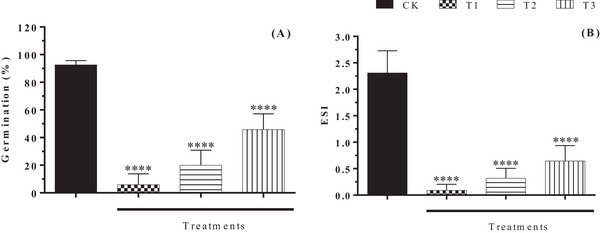
Germination percentage (A) and emergence speed index (ESI) of *Handroanthus impetiginosus* seedlings (B) subjected to different concentrations of leaf litter extracts from *Calotropis procera* Germination percentage: CK: 92.5%; T1: 5.8%; T2: 20%; T3: 42% (average). One‐way analysis of variance (ANOVA). Mean (±standard deviation). ****p < 0.0001 compared to the control. CK: control; T1: 9 g/kg; T2: 18 g/kg; T3: 27 g/kg.

The germination of *H. impetiginosus* was negatively affected by the leaf decomposition of *C. procera* at all concentrations tested (T1, T2, and T3), with the greatest re‐duction in germination observed in T1, where only 5.83% germinated (Figure 3A). A similar result was observed in the study by Al‐Rowaily [53] where the effect of the volatile oil of *C. procera* affected the germination of *Bidens pilosa* L. showing a reduction of 91.7% in the germination rate compared to the control group. The compounds phytol (C_20_H_40_O) and linoleic acid (C_20_H_36_O_2_) were observed in both studies. Al‐Rowaily [53] associated these compounds with the results observed in their study. The emergence speed index was also negatively affected by all three treatments (Figure 3B). The results were most evident at concentrations of 9 g/kg (0.08 ± 0.11), followed by 18 g/kg (0.32 ± 0.18), and 27 g/kg (0.64 ± 0.29).

The action of substances is not completely specific, and their effects can vary depending on the translocation process and concentration, where the same substance can have different functions on receptor species [75]. In the present study, the most evident results in germination processes (germination and ESI) were observed at the lowest concentrations of leaf litter extracts evaluated, highlighting that depending on the concentration of compounds present in the environment, the result can be more or less significant in the germination processes.

In the study by Silva [42], a negative result was observed for the emergence index of *Myracrodruon urundeuva* Allemão subjected to the leaf litter of *A. indica* A. Juss during 3 months of decomposition. In both studies, the presence of compounds from the terpene class prevailed, which in practice may indicate their interference with the germination of the receptor species. Al‐Rowaily [53] also attributed the results found in their study to the presence of terpenoids in the volatile oil of *C. procera*, with negative results observed in the germination processes of the species *B. pilosa* L. and *Dactyloctenium aegyptium* (L.) Willd.

Regarding the time required for the emergence of 50% of *H. impetiginosus* seedlings, a negative response was observed in all three treatments evaluated (T1, T2, and T3) (Table 4). It was found that the seedlings subjected to the highest concentration of leaf litter experienced a greater delay in the emergence process (17.30 ± 2.53), compared to the control group (10.02 ± 1.60). In the study by Almeida‐Bezerra [43], the allelopathic potential of *Mesosphaerum suaveolens* (L.) Kuntze litter on the growth of typical Cactaceae species from the Seasonally Dry Tropical Forest was evaluated. An increase in the *T*_50_ (%) of the seedlings from two of the evaluated species was observed, with more significant results at higher litter concentrations. Among the nine constituents observed in *M. suaveolens* there were three terpenoids (C_30_), which may have influenced the observed allelopathic outcome.

**TABLE 4:** Allelopathic activity of leaf litter extracts from *Calotropis procera* on the synchrony and the time for 50% of seeds to germinate (*T*_50_) of *Handroanthus impetiginosus*.

Parameter	Treatments			
	CK	T1	T2	T3
Synchrony	0.16 ± 0.10	0.07 ± 0.15^ns^	0.11 ± 0.13^ns^	0.06 ± 0.06^ns^
*T*_50_ (%)	10.02 ± 1.60	16.50 ± 2.30^*^	15.37 ± 3.14^*^	17.30 ± 2.53**

*Note*: CK: control; T1: 9 g/kg; T2: 18 g/kg; T3: 27 g/kg. One‐way analysis of variance (ANOVA).

Abbreviation: ns, no statistical significance.

^*^ *p* < 0.05, **p < 0.01 compared to the control (mean ± standard error).

The synchrony of the germination process of *H. impetiginosus* seedlings was not statistically influenced by any leaf litter concentrations tested (Table 4), compared to the control group (CK). Allelochemicals can affect species differently, possibly exhibiting varied levels of tolerance, thereby avoiding certain mechanisms of action or levels of phytotoxicity [76, 77].

### 2.4 Development Test

The litter from *C. procera* had a positive effect on the average length of *H. impetiginosus* radicle in treatment T2 (138.60 ± 16.43 mm), compared to the control group (105 ± 21.30 mm). Regarding the aboveground part of the young plants, only the epicotyl showed significant growth in average length, with positive results for all evaluated treatments (T1, T2, and T3). The concentration of 18 g/kg (72.70 ± 12.90 mm) showed the most pronounced result, followed by 9 g/kg (69.30 ± 15.93 mm) and 27 g/kg (62.50 ± 10.59 mm).

Allelopathic activity is generally observed in the early stages of growth and development of plant species, which may explain the results observed for the germination process and development of the aboveground (epicotyl and hypocotyl) and underground (root) parts of young *H. impetiginosus* plants subjected to *C. procera* litter (Table 5). Huang [78] reported that the litter from *Cinnamomum septentrionale* Hand. Mazz negatively affected the initial growth phase of *Eucalyptus grandis* Hill ex Maid, but after 5 months, the growth rate (height) was accelerated compared to the control group (CK). As the leaf decomposition process progresses, the amount of biomass tends to degrade and gradually decrease due to the action of soil microorganisms. As a result, the compounds are transformed into nutrients available to the plant community [79, 80]. This process may clarify the results seen in the development parameters and average growth of the species studied in this research, as well as in Huang [42], in which during the final stages of the decomposition phase, the receiving species exhibited greater height growth.

**TABLE 5:** Allelopathic activity of *Calotropis procera* leaf litter on the root, epicotyl, and hypocotyl length of *Handroanthus impetiginosus*.

Treatment	Root	Epicotyl	Hypocotyl
	Length (mm)		
Control (CK)	105 ± 21.30	46.20 ± 9.83	12.60 ± 2.75^ns^
T1	118 ± 22.11^ns^	69.30 ± 15.93**	14.60 ± 3.50^ns^
T2	138.60 ± 16.43**	72.70 ± 12.90***	15.40 ± 2.75^ns^
T3	117.40 ± 10.24^ns^	62.50 ± 10.59^*^	13.60 ± 3.13^ns^

*Note*: CK: Control; T1: 9 g/kg; T2: 18 g/kg; T3: 27 g/kg. One‐way analysis of variance (ANOVA). Unit of measurement: mm.

Abbreviation: ns, not statistically significant.

^*^ *p* < 0.05, **p < 0.01, and ***p < 0.001 compared to control (mean ± standard error).

The leaf litter of *C. procera* positively affected the dry biomass of *H. impetiginosus* (Table 6) in all three treatments evaluated (T1, T2, and T3), varying according to the plant organ (root, epicotyl, and hypocotyl). The epicotyl showed the most significant response. The 18 g/kg treatment (T2) demonstrated the highest significance (0.40 ± 0.08 g). The observed results for dry biomass reflect the significant growth seen in Table 5, where the young plants in the T2 treatment exhibited the highest relative growth.

**TABLE 6:** Allelopathic activity of *Calotropis procera* leaf litter on the root, epicotyl, and hypocotyl dry biomass of *Handroanthus impetiginosus*.

Treatments	Root	Epicotyl, dry biomass (g)	Hypocotyl
	Dry biomass (g)		
Control (CK)	0.12 ± 0.04	0.12 ± 0.04	0.01 ± 0.007
T1	0.19 ± 0.05^*^	0.35 ± 0.09****	0.02 ± 0.01^ns^
T2	0.13 ± 0.04^ns^	0.40 ± 0.08****	0.02 ± 0.008**
T3	0.12 ± 0.03^ns^	0.34 ± 0.09****	0.02 ± 0.008^ns^

*Note*: CK: Control; T1: 9 g/kg; T2: 18 g/kg; T3: 27 g/kg. One‐way analysis of variance (ANOVA). Unit of measurement: g.

Abbreviation: ns, not statistically significant.

^*^ *p* < 0.05, **p < 0.01, and ***p < 0.001 compared to control (mean ± standard error).

The action of bioactive compounds associated with the decomposition of organic matter depends on the time the biomass is incorporated into the soil, it is degraded by chemical and/or microbial activity in the soil [81]. In the study by Sodaeizadeh [77], the effect of *Peganum harmala* L. decomposition on *Avena sativa* L. and *Convolvulus arvensis* L. was evaluated, and a negative response was observed in the parameters assessed during the first days of organic matter incorporation into the soil, but after 7 days, no evident response was detected in the development of the receptor species. The results found for the biomass of *H. impetiginosus* may be a response to the additional nutrients added to the soil observed for all concentrations evaluated (T1, T2, and T3), being associated with the degradation of *C. procera* allelochemicals by microbial activity (Table 1).

The chlorophyll pigment content of *H. impetiginosus* did not show any statistical difference at any concentration of *C. procera* leaf litter when compared to the control (CK) (Table 7). These results suggest that even with the increase in organic matter and the decrease in allelochemicals distributed in the soil, there was no significant change in the photosynthetic process associated with the young plants of the receptor species.

**TABLE 7:** Allelopathic activity of *Calotropis procera* leaf litter on the chlorophyll and carotenoid content of *Handroanthus impetiginosus*.

Treatment	Chlorophyll *a*	Chlorophyll *b*	Chlorophyll total	Carotenoids
	Absorbance (nm)			
Control (CK)	0.16 ± 0.22	0.02 ± 0.48	0.28 ± 0.27	0.49 ± 0.34
T1	0.18 ± 0.05^ns^	0.05 ± 0.29^ns^	0.36 ± 0.27^ns^	0.31 ± 0.23^ns^
T2	0.15 ± 0.09^ns^	0.02 ± 0.22^ns^	0.34 ± 0.25^ns^	0.32 ± 0.32^ns^
T3	0.30 ± 0.01^ns^	0.35 ± 0.37^ns^	0.31 ± 0.11^ns^	0.52 ± 0.31^ns^

*Note*: CK: Control; T1: 9 g/kg; T2: 18 g/kg; T3: 27 g/kg. One‐way analysis of variance (ANOVA). Unit of measurement: nm.

Abbreviation: ns, not statistically significant.

^*^ *p* < 0.05 compared to control (mean ± standard error).

**FIGURE 4:**
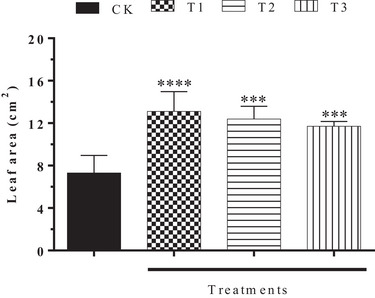
Allelopathic effect of the decomposition of *Calotropis procera* leaf litter on the leaf area of young *Handroanthus impetiginosus* individuals. CK: Control; T1: 9 g/kg; T2: 18 g/kg; T3: 27 g/kg. One‐way analysis of variance (ANOVA). ns, NOT statistically significant; ***p < 0.001; ****p < 0.0001 compared to control (mean ± standard error). Unit of measurement: cm^2^.

In addition to the results observed for the biomass of *H. impetiginosus*, the leaf litter of *C. procera* caused an increase in leaf area, demonstrating that the presence of nutrients in the soil positively influenced different plant organs of the receptor species (Figure 4). These results are evidenced in Figure 4, where the lowest concentration of leaf litter (9 g/kg) showed the most significant result (13.11 ± 1.87 cm^2^) compared to the control group (7.31 ± 1.64 cm^2^). When we associate the results for the treatments with the leaf litter added to the soil, we observe that the lowest leaf concentration (9 g/kg) showed a leaf area of 13.11 ± 1.87 cm^2^, followed by 12.38 ± 1.20 and 11.71 ± 0.44 cm^2^ (18 and 27 g/kg, respectively). This result is associated with the longer persistence of allelochemicals in the soil, resulting from the greater amount of available biomass.

### 2.5 Morphoanatomical Changes

**FIGURE 5:**
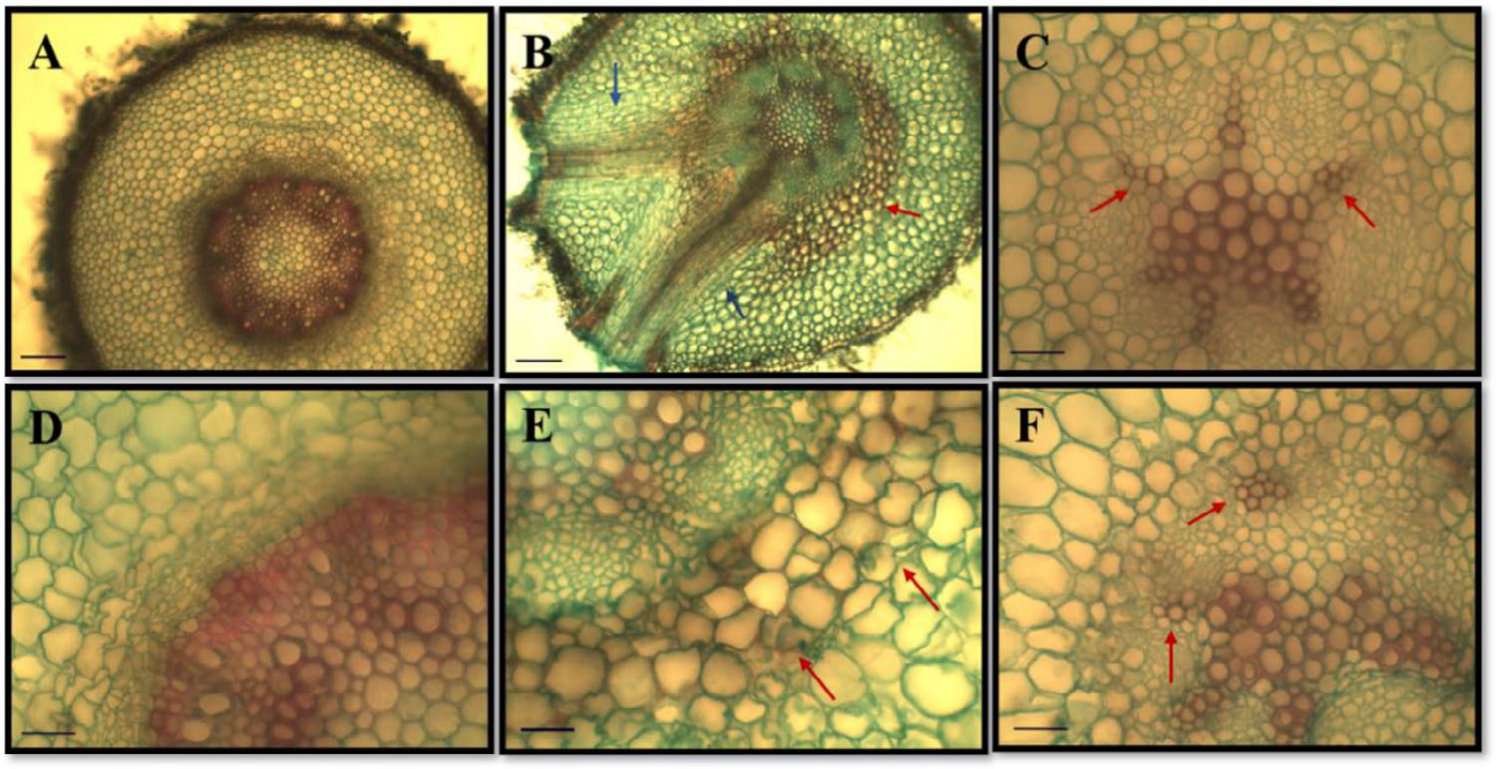
Morphoanatomical aspects of the radicle of *Handroanthus impetiginosus* in cross‐sections, subjected to different concentrations of *Calotropis procera* leaf litter. (A, D) Radicle of seedling from the control group; (B, E) radicle of seedling subjected to the 9 g/kg treatment (T1) showing presence of dark content cells (red arrow) and pronounced development of secondary roots (blue arrow); (C) radicle of seedling subjected to the 18 g/kg treatment (T2) showing reduction in the vascular system (red arrow); (F) radicle of seedling subjected to the 27 g/kg treatment (T3) with delayed development of the vascular system (red arrow). Scale bar = 300 µm (A, B), 90 µm (C, F).

Regarding morphoanatomical analysis, a significant variation in tissue thickness was observed in the radicle and young stems of *H. impetiginosus* exposed to different concentrations of *C. procera* leaf biomass (Table 8). The young stems showed a positive variation in all evaluated regions, with greater significance in the cortex and vascular system. Treatments 9 g/kg (T1) and 18 g/kg (T2) exhibited a stronger response in tissue thickening. The radicle of *H. impetiginosus* showed a significant response only in the vascular system, with a decrease in associated tissue thickness (Table 8). The treatment with 27 g/kg showed the greatest significance (333.89 ± 15.17 µm) (Figure 5F), followed by 18 g/kg with an average of 333.58 ± 19.72 µm, compared to the control group (423.18 ± 51.11 µm) (Figure 5C).

**TABLE 8:** Tissue thickness of different regions of young stems and radicle of *Handroanthus impetiginosus* in cross‐section, subjected to different concentrations of *Calotropis procera* leaf litter.

Organ	Variable	Control (CK)	T1	T2	T3
		Thickness (µm)			
Young stems	Epidermis	37.44 ± 3.06	43.47 ± 1.93^*^	45.94 ± 1.17^**^	47.38 ± 1.50^**^
	Cortex	954.91 ± 66.66	1805.01 ± 170.27^***^	1752 ± 137.23^***^	1901 ± 155.82^***^
	Vascular system	481.05 ± 26.14	802.48 ± 47.53^****^	807.16 ± 7.30^****^	749.98 ± 57.99^***^
Radicle	Epidermis	39.74 ± 1.87	40.13 ± 3.75^ns^	39.28 ± 3.88^ns^	45.44 ± 3.21^ns^
	Cortex	798.14 ± 99.08	890.07 ± 119.49^ns^	774.48 ± 56.79^ns^	870.40 ± 124.21^ns^
	Vascular system	423.18 ± 51.11	377.16 ± 24.52^ns^	333.58 ± 19.72^**^	333.89 ± 15.17^***^

*Note*: CK: Control; T1: 9 g/kg; T2: 18 g/kg; T3: 27 g/kg. One‐way analysis of variance (ANOVA). Unit of measurement: µm.

Abbreviation: ns, no statistical significance.

^*^ *p* < 0.05, **p < 0.01, ***p < 0.001, ****p < 0.0001, compared to the control (mean ± standard error).

Regarding qualitative analysis, a delay in the development of conducting tissues was observed, where the control (CK) showed well‐established secondary growth (Figure 7A), compared to treatments containing *C. procera* leaf litter (T1, T2, and T3). For the concentration (T1), initial secondary growth was observed, but to a lesser extent compared to the control group (CK), whereas in the other treatments (T2 and T3), seedlings exhibited primary growth of conducting tissues. These results are consistent with the thickness data of the vascular system tissues, which showed greater significance in the last two treatments (18 and 27 g/kg) (Table 8).

Pina [82] evaluated the allelopathic potential of the aqueous extract of *Eugenia dysenterica* DC. on *Sesamum indicum* L., observing a decrease in length and an increase in average radicle diameter. This result was similar to what was observed in our study for *H. impetiginosus*, where the seedlings had shorter young stems with larger diameters (Table 8). The effect of the leaf decomposition of *C. procera* on the morphoanatomy of *H. impetiginosus* is shown in Figure 5. Qualitative analysis revealed morphoanatomical variations in the 9 g/kg treatment (T1), with the presence of dark content in cells, indicating the presence of phenolic compounds (a type of secondary metabolite, precursor to lignin synthesis), which act as chelators (Figure 5B,E). The endodermis primarily functions to regulate the transport of water and nutrients from the cortex to the vascular system, and it includes a band of cells whose walls develop Casparian strips, serving as a barrier that prevents apoplastic transport (through cell walls) in plants with primary growth [83]. Tissue darkening or “burning” may represent a strategy to immobilize metabolites in the endodermis region, preventing further damage to adjacent tissues.

Another qualitative alteration observed in the T1 treatment (9 g/kg) was the parenchymal cells in the middle cortex region showing plasmolysis and hyperplasia, displaying irregular and uneven shapes compared to the control group (CK) (Figure 5B). In the study by Ximenez [84], irregularity in cortical cells of *Euphorbia heterophylla* Müll. Arg. was also observed under the influence of crude extract from *Machaerium hirtum* (Vell.) Stellfeld, with results attributed to the presence of compounds from the donor species, including Lupeol (C_30_H_50_O) [85], identified in the phytochemical analysis of our study (Tables 2 and 3). As shown in Figures 5C and 4F, significant variation in vascular system thickness in radicles was observed only in treatments 18 g/kg (T2) and 27 g/kg (T3), respectively. These findings may indicate that at the T1 concentration, seedlings attempted to immobilize allelochemicals near the endodermis region to prevent translocation through the vascular system, but at higher concentrations (18 and 27 g/kg), it reflected in delayed seedling development.

**FIGURE 6:**
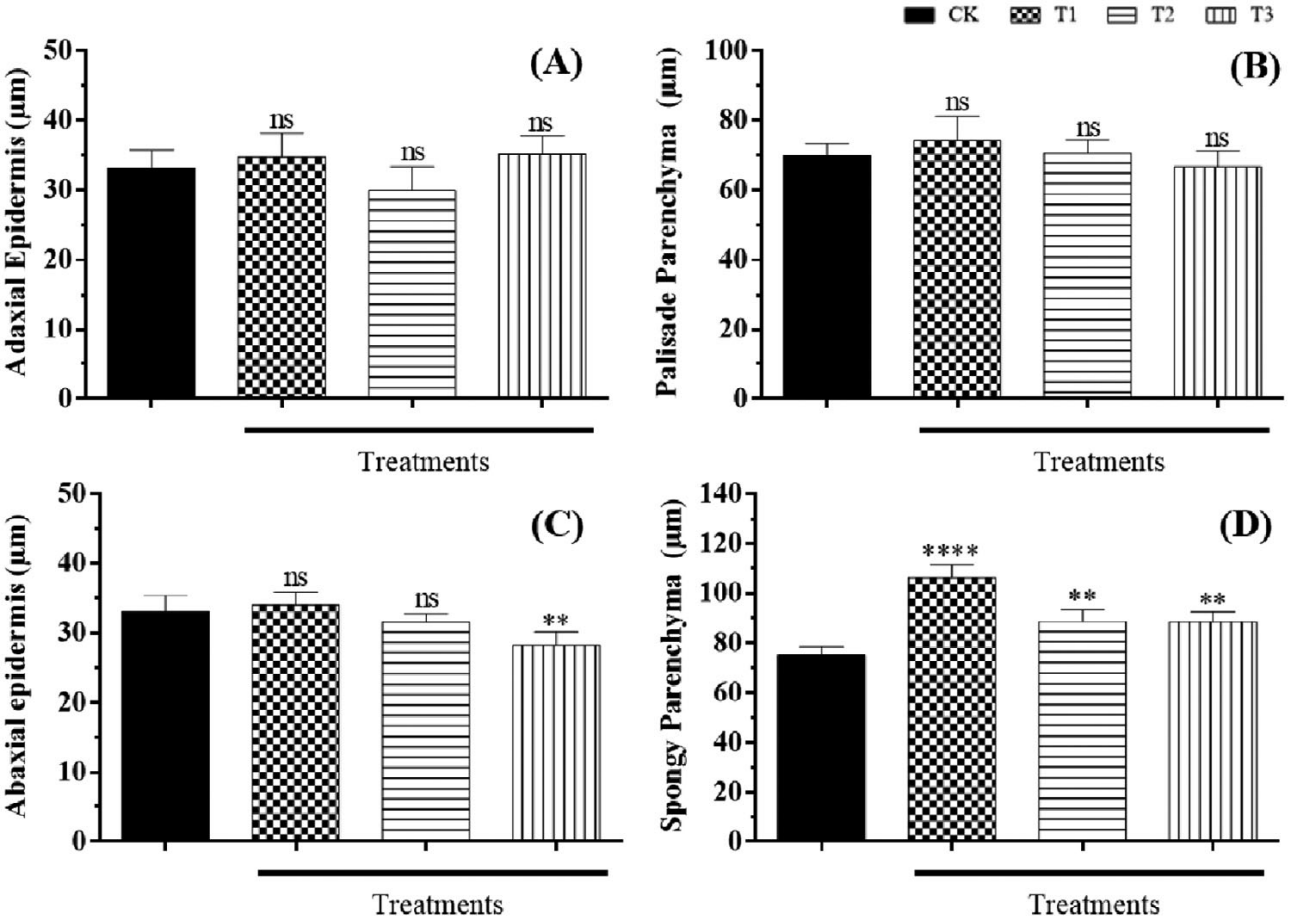
Allelopathic effect of *Calotropis procera* leaf litter decomposition on the thickness of tissues and regions of *Handroanthus impetiginosus* leaves in cross‐section. (A) Adaxial epidermis; (B) palisade parenchyma; (C) abaxial epidermis; (D) spongy parenchyma. CK: Control; T1: 9 g/kg; T2: 18 g/kg; T3: 27 g/kg. One‐way analysis of variance (ANOVA). Mean (±standard deviation). ns, not statistically significant; **p < 0.01; ****p < 0.0001, compared to control. Unit of measurement: µm.

Regarding the formation of secondary roots, an increase was observed in the 9 g/kg treatment (T1) (Figure 6B), with no lateral roots present in the control group (CK). This type of alteration has already been reported in other studies associated with the allelopathic and phytotoxic potential in the roots of recipient seedlings [82, 86, 87]. Hormonal imbalances can lead to the early formation of lateral roots, mainly associated with low levels of ethylene/auxin, hormones that are important in the formation of these structures [88]. Allelochemicals can act on different physiological mechanisms in plants, including hormonal pathways, accelerating or delaying the development of certain structures, such as secondary roots [89, 90]. In a study by Alcântara [87], the development of lateral roots, a decrease in length, and an increase in the diameter of *L. sericeus* seedlings were also observed when subjected to the crude extract of *Commelina benghalensis*, demonstrating that the metabolites have similar pathways of action regardless of the donor species used. In their study, the authors also associated alterations in hormonal pathways as a response to the early development of secondary roots.

In general, the products resulting from the foliar decomposition of *C. procera* did not cause significant changes in the thickness of *H. impetiginosus* leaf tissues at different concentrations (Figure 6), except for the spongy parenchyma (Figure 6D) and the adaxial epidermal cells (Figure 6B). The spongy parenchyma showed greater thickness in the 9 g/kg treatment (T1), with an average of 106.21 ± 5.31 µm. Treatments T2 (18 g/kg) and T3 (27 g/kg), although not differing from each other, increased the thickness of the spongy parenchyma compared to the control, measuring 88.47 ± 4.92 and 88.35 ± 4.16 µm, respectively. The thickness of the abaxial epidermal cells showed a significant response only for treatment T3 (28.26 ± 1.92 µm), compared to the control group (33.06 ± 2.31 µm).

**FIGURE 7:**
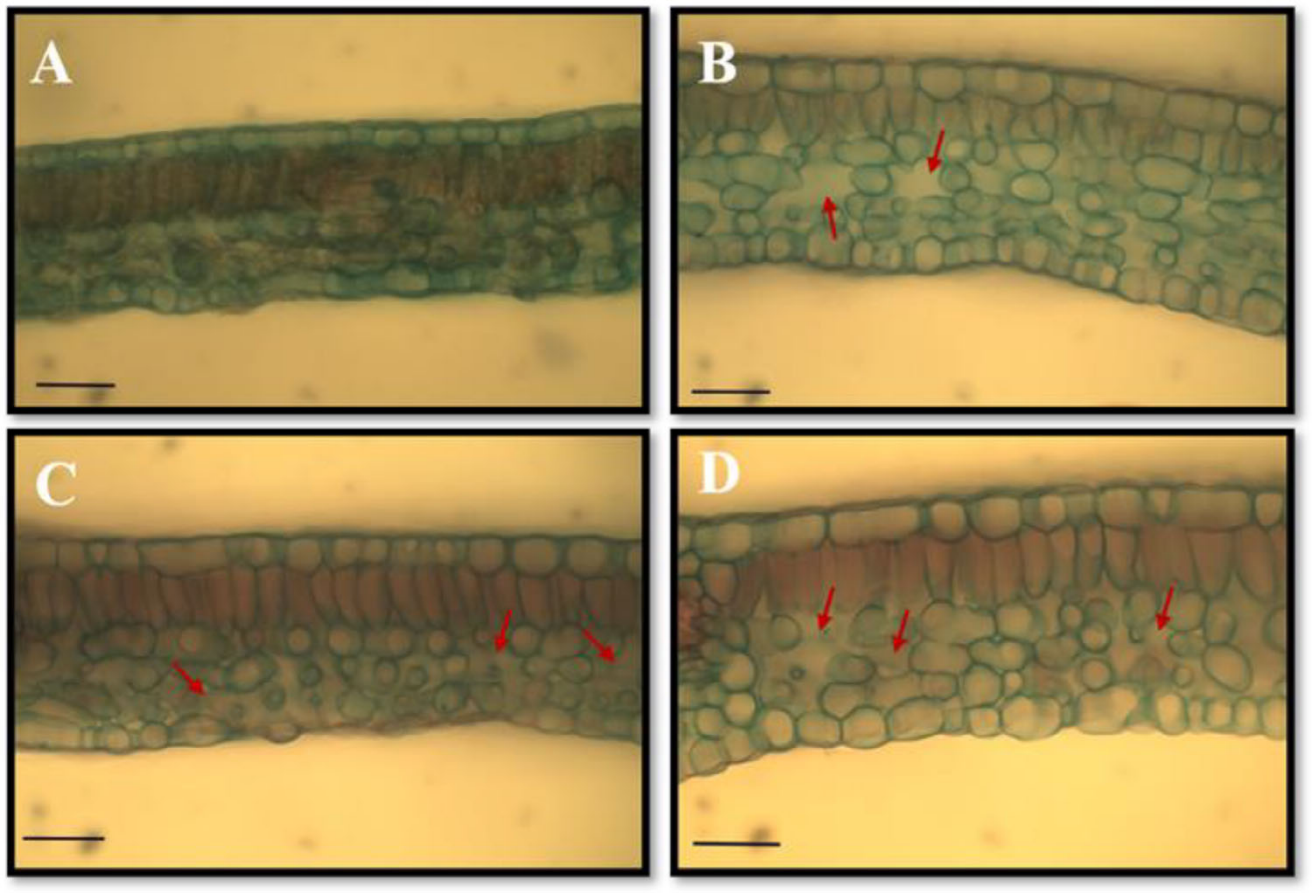
Morphoanatomical aspects of the leaves of *Handroanthus impetiginosus* in cross‐sections, subjected to different concentrations of *Calotropis procera* leaf litter. (A) Leaf of seedling from the control group (CK); (B) leaf of seedling subjected to the 9 g/kg treatment (T1), showing an increase in intercellular space in the spongy parenchyma (red arrow); (C) young leaf subjected to the 18 g/kg treatment (T2) showing an increase in intercellular space in the spongy parenchyma (red arrow); (D) young leaf subjected to the 27 g/kg treatment (T3) showing an increase in intercellular space in the spongy parenchyma (red arrow). Scale bar = 100 µm (A–D).

The morphoanatomical analysis of the leaf mesophyll of *H. impetiginosus* showed an increase in intercellular spaces in the spongy parenchyma across all three treatments (Figure 7). These results can explain the positive variations in spongy parenchyma thickness observed in Figure 6D, indicating a plant strategy to maximize light absorption and improve its direction, enhancing the capture by the chloroplasts [91]. Some authors have attributed variations in leaf mesophyll thickness as indicative of sensitivity to external stresses, such as pollutants and contaminants [92, 93, 94, 95]. Recent studies have shown that variations in the thickness of the spongy parenchyma in leaves can represent a plant response to stress and adverse conditions, where the increase in intercellular space favors CO_2_ accumulation and aids in photosynthetic processes [95, 96, 97].

The cells on the abaxial side of the *H. impetiginosus* leaf showed a negative response in tissue thickening at the 27 g/kg concentration (Figure 7F), with an average of 28.26 ± 1.92 µm, compared to the control group (33.06 ± 2.31 µm). Other authors have also observed variations in epidermal thickness when exposed to different types of stress, which can be a superficial indicator of the response of the plants to external pressure [93, 98].

**FIGURE 8:**
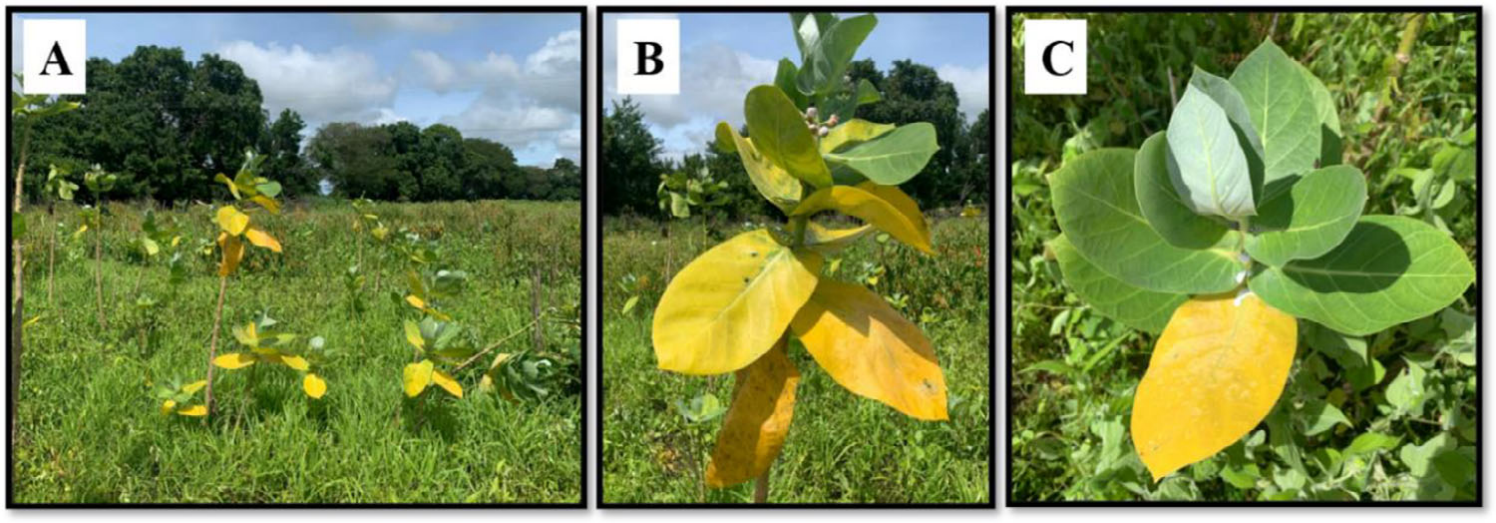
*Calotropis procera* (Aiton) W.T. Aiton in senescing stage, Missão Velha City—CE, Brazil. (A) Plants in Caatinga area. (B) Mature plant displaying its initial and final phases of leaf senescence. (C) Young plant with leaves in the initial phase of senescence.

The qualitative results observed in young plants of *H. impetiginosus* demonstrate the allelopathic potential of *C. procera* litter at different concentrations and in various plant organs (root, stem, and leaf). The increase in intercellular spaces (Figure 7) or the early development of secondary roots (Figure 8) could represent a strategy by the plant to mitigate damage associated with allelochemicals from the donor species. The additional formation of trichomes and lateral roots in cases of phytotoxicity is a plant strategy to increase the water absorption area and reduce phytotoxic effects. Depending on the concentration, the effects may be more or less significant, resulting in intense or mild thickness of the internal tissues of young plants (Table 8).

## 3 Conclusion

The germination and post‐germination processes in *H. impetiginosus* indicate an allelopathic effect associated with the chemical constituents (fatty acids, diterpenes, triterpenes, and steroids) released during the leaf decomposition process of *C. procera*. The dose‐dependent results with greater significance at lower concentrations of leaf litter demonstrate that some allelochemicals have more specific effects depending on the concentration.

The negative allelopathic action of *C. procera* on the germinability, germination rate, and emergence speed index of *H. impetiginosus* can negatively impact its vegetative succession process. This indicates the importance of controlling and managing the donor species, as its introduction into natural environments could contribute to the reduction or extinction of native species in the medium and long term.

The results obtained in a controlled environment related to height growth, biomass, and leaf area indicate that the allelochemicals present in the substrate decrease with decomposition time. However, in the natural environment, since the donor species tends to continue releasing leaf litter and chemical constituents through decomposition, the results may differ. Allelochemicals from the leaf litter decomposition process of *C. procera* caused significant variations in the morphoanatomy and physiology of the seedlings (young stems and radicles) and young leaves of *H. impetiginosus*.

This study is relevant to the fields of ecology and plant production, as it enhances our understanding of the processes involved between invasive and native species in natural environments. It is also crucial for the agricultural sector, which is directly affected by biological invasions of weeds.

## 4 Experimental Section

### 4.1 Collection of Botanical Material

**FIGURE 9:**
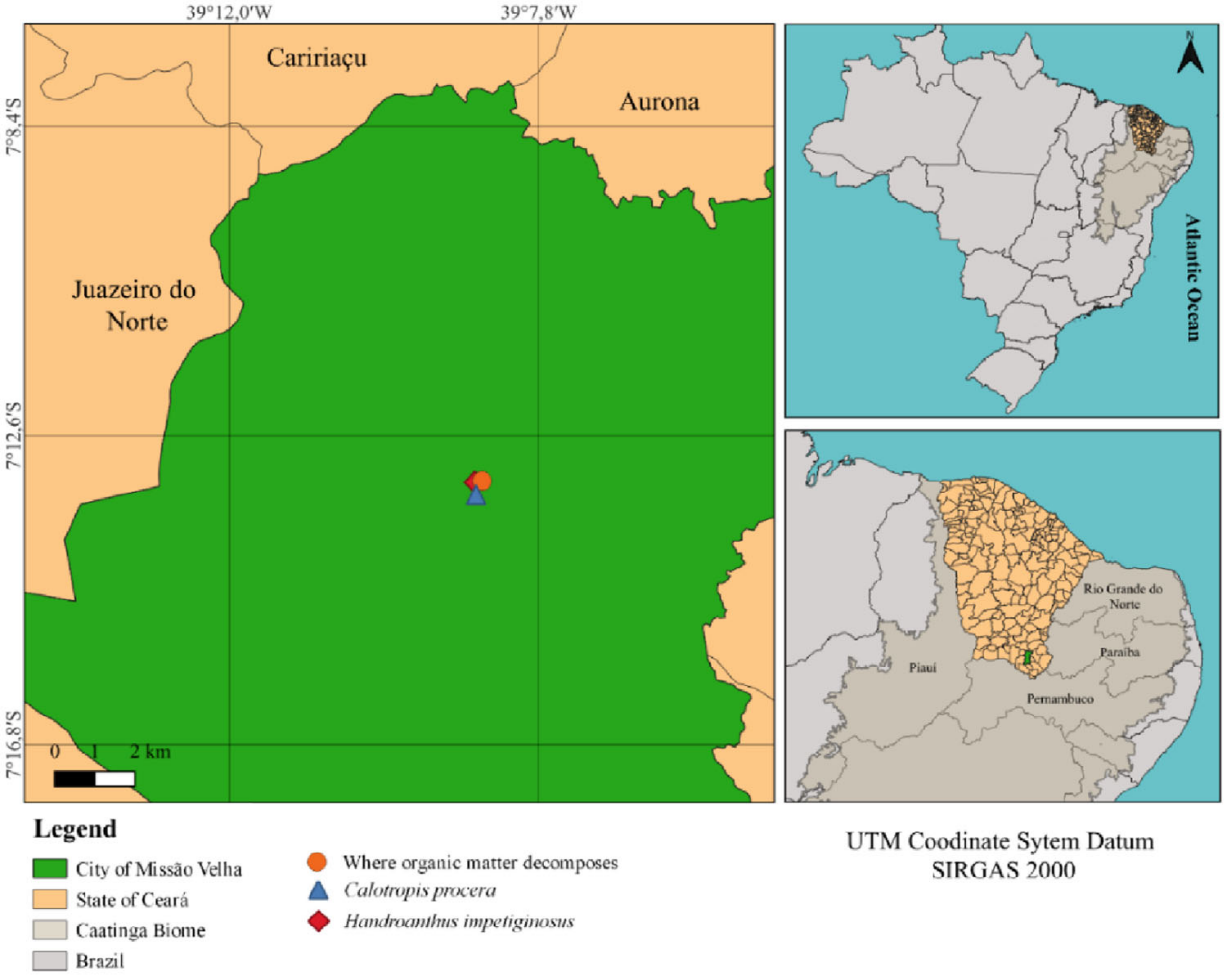
Map of the collection sites of *Calotropis procera* (Aiton) W.T. Aiton and *Handroanthus impetiginosus* (Mart. ex DC.) Mattos and leaf decomposition assay collection site. Missão Velha City—CE, Brazil.

Reproductive branches (flowering and/or fruiting) of *H. impetiginosus* (recipient species) (7°13′14″ S and 39°8′40″ W) and *C. procera* (donor species) (7°13′24″ S and 39°8′39″ W) were collected in an area of Caatinga (Northeast Brazil) in the city Missão Velha—Ceará State, Brazil (Figure 9). Once identified, the botanic material was deposited in the collection of the Herbarium Caririense Dárdano de Andrade‐Lima—HCDAL, of the Universidade Regional, do Cariri—URCA, under the numbers 15 991 and 15 993, respectively.

Senescing leaves of *C. procera* were collected for allelopathy assays in September 2022 (dry period) (Figure 8). The criteria for collection were based on sampling the leaves that underwent natural plant senescence, or the ones that displayed a completely yellowish color and could be detached from the adult plant without the presence of exudation. The seeds of *H. impetiginosus* that were used in the germination bioassays were collected in the same area as *C. procera* leaves, aiming to maintain the natural conditions of occurrence of both species (Figure 9). The seeds were sampled in August 2022, and kept in paper bags and under refrigeration (10°C) until the tests were carried out.

### 4.2 Soil Collections

The soil used in the leaf decomposition process was collected from Horizon A (10–20 cm). Based on the characteristics of macro‐ and micronutrients, the soil in the area was classified as sandy, due to its low fertility and moderate acidity. The soil has a reddish color, possibly related to the presence of iron oxides (Fe^3+^), a characteristic typical of well‐drained tropical soils exposed to weathering in semiarid regions [99]. The collection site was in the area of incidence of the recipient species used in the study, aiming not to alter its natural germination conditions. The soil was collected during the dry season, in September 2022, the same period as the collection of *C. procera* leaves, also simulating the same natural environmental conditions. As for meteorological data, the average temperature during the decomposition months (October, November, and December) ranged from 28.35°C to 29.03°C. Relative humidity during this period ranged from 51.52% to 60.98%, characterizing it as a dry season [100].

### 4.3 Leaf Decomposition

To simulate the decomposition process, *C. procera* leaves were mixed into the soil in alternating layers and left to decompose in different concentrations of total soil mass (w/w) for 90 days, according to a methodology adapted from [42, 43], in fine mesh bags to allow the passage of moisture and light necessary for the natural decomposition of biomass. The amount of leaves added was based on the annual leaf production of the species, estimated at approximately 9 t/ha/year [101], equivalent to 9 g/kg of leaves established for the first concentration of the experiment, which corresponds to an initial concentration of 9 g of leaves per kg of soil. The concentrations tested were: 9 (T1), 18 (T2), and 27 (T3) g/kg of Caatinga soil [42, 43]. A control group (CK) was established containing only Caatinga soil, without the addition of biomass. The samples were placed in a 5 m^2^ area of native Caatinga vegetation, located in the municipality of Missão Velha, Ceará, Brazil, where they remained exposed to natural environmental conditions throughout the experimental period.

### 4.4 Analysis of Soil Physicochemical Parameters

After leaf decomposition, all soil samples (CK, T1, T2, and T3) were analyzed in terms of physicochemical parameters: pH, macro‐ and micronutrients, base saturation, CEC, and aluminum saturation (%). The tests were carried out at the Soil Fertility Laboratory at Agronomic Institute of Pernambuco—IPA (Recife—PE), following EMBRAPA methodology [102].

### 4.5 Phytochemical Analysis

#### 4.5.1 Preparation of Extracts

The extraction of compounds was carried out at a concentration of 27 g/kg, corresponding to the treatment (T3). The extraction was performed immediately after the decomposition period, with the soil free of leaf particles. The soil from the control group (CK) was also subjected to the same procedure. The phytochemical analysis was carried out only on the highest concentration of *C. procera* litter, since for all concentrations tested, a variation is expected only in the abundance of allelochemicals, maintaining the chemical composition.

To obtain the organic extracts it was followed the methodology proposed by Huang [78]. Initially, the nonpolar compounds present in the soil were extracted through exhaustive extraction using 8 L of *n*‐hexane and 4 kg of soil (1:2 w/v), for 96 h in the dark. After filtration, the soil was left to dry at room temperature and subjected to another extraction using ethanol for the same period, to obtain polar compounds. After filtering, the solvent was distilled using a roto evaporator (model Q‐344B, Quimis, Diadema, Brazil).

#### 4.5.2 GC/MS Analysis

The crude extracts of both solvents (hexane and ethanol) were weighed on an analytical scale and subsequently diluted in their respective solvent (1:1 w/v). Subsequently, an aliquot of 50 µL was transferred to inserts and dried in a vacuum sample concentrator. The extracts were derivatized with 25 µL of pyridine and 25 µL of N,O‐bis‐(trimethylsilyl)‐trifluoroacetamide (BSTFA) for 1 h at 70°C. Samples were injected (1 µL) into a gas chromatogram (model 6850 Network GC System, Agilent) coupled to mass spectrometry (model 5975 VL MSD, Agilent) (GC/MS), equipped with an Agilent HP5‐MS capillary column (30 m, 0.25 mm, 0.25 µm). The initial column temperature was set at 100°C for 5 min, and increased at a rate of 5°C/min to a final temperature of 320°C, totaling a run time of 49 min. Helium was the carrier gas at 1 mL/min. The injection, ion source, and quadrupole temperatures were set at 300°C, 230°C, and 150°C, respectively. Ionization was by electron impact (70 eV), working in full scan acquisition mode between 50 and 600 *m*/*z* at 2.66 scans.

The mass data obtained for each extract went through the processes of deconvolution, peak alignment, and calculation of the linear retention index (LRI)—using a standard of C8 to C40 alkanes. Compound annotations were made using suggestions given by GNPS and the NIST library, generating a table of characteristics composed of peak area, molecular ion, retention time, and a possible suggestion for each detected compound. A minimum cosine index of 0.30 was determined and an LRI window of 30 was applied to identify the compounds. Compounds with a cosine index above the minimum but without LRI matching were considered to be from the same suggestion class obtained by the GNPS data [103].

### 4.6 *H. impetiginosus* Germination and Development Test

After the leaf decomposition period, the bags containing the soil with different concentrations of litter were taken to the Applied Botany Laboratory (LBA), Universidade Regional do Cariri—URCA, Crato—CE, Brazil, to carry out the trials of seed germination and development of *H. impetiginosus*.

#### 4.6.1 Germination Test

The seeds used as recipients were disinfected with a 5% sodium hypochlorite solution for 5 min and subsequently washed in distilled water for the same time [104]. Germination bioassays were carried out in a greenhouse, in polyethylene trays with 200 cells. The soil containing the decomposed plant material of *C. procera* was used as substrate, together with the control soil (CK). For the germination test, 30 seeds were used per repetition, with each seed allocated to an individual cell. Each tray containing the 30 seeds constituted one experimental repetition, and four repetitions were performed for each treatment (quadruplicated) and control group. (CK). Each cell was moistened with 7 mL of distilled water and then received a seed. Watering was carried out daily to maintain substrate humidity [4]. Daily observations were carried out for 15 days. The seeds that showed the emergence of the hypocotyl were considered germinated.

At the end of each evaluation, germinability (%), emergence speed index (ESI), synchrony (E), and germination time of 50% of seeds (*T*_50_) were calculated. To calculate the germination percentage of the seeds of the receiving species, the formula was used: *G* = (*N*/*A*) × 100, where *G* is the germinability; *N* is the number of germinated seeds; *A* is the total number of seeds evaluated [105]. The ESI was calculated according to Maguire using the formula: ESI = *E*_1_/*N*_1_ + *E*_2_/*N*_2_+…*E_n_*/*N_n_*, where *E*_1_, *E*_2_, and *E*_n_ represent the number of normal seedlings that emerged, recorded in the first, second, and last counting, respectively; and *N*_1_, *N*_2_, and *N_n_*, the number of days from sowing to the first, second, and last count. To determine the time needed to reach 50% germination (*T*_50_), it was used the formula proposed by Coolber [106]: *T*_50_ = *t_i_* + (*N*/2 − *n_i_*)*(*t_j_* − *t_i_*)/(*n_j_* − *n_i_*), where *N* is the final number of germinated seeds; *n_j_* and *n_i_* are accumulated number of seeds germinated by time counts (*t_j_* and *t_i_*), respectively, where *n_i_* < N/2 < *n_j_*.

#### 4.6.2 Seedlings Development Test

To investigate the effects of the metabolites released in the soil from *C. procera* leaf decomposition on the growth of *H. impetiginosus*, seeds of this plant were placed to germinate in disinfected gerboxes containing two sheets of germitest paper as a substrate, moistened with 5 mL of distilled water. The bioassays were placed in a biochemical oxygen demand (BOD) chamber with a constant temperature of 30°C and a 12‐h photoperiod (light/dark) [87]. After germination, the seedlings that showed 10 mm of radicular protrusion were transplanted into polyethylene trays with 30 cells (200 mL), containing the soils in different litter concentrations (T1, T2, T3) and the control soil (CK). Before sowing, the soil was moistened with distilled water, and the volume was determined based on the field capacity of the soil [4].

A total of 30 replications were set for each treatment, and each replication consisted of one *H. impetiginosus* seedling. After sowing, the containers were taken to the greenhouse where they remained for 2 months (60 days). During the experiment, watering was carried out every two or three days according to the requirements to keep the relative humidity of the soil. After 60 days, the seedlings of the recipient species were taken to the laboratory, removed from the containers, and carefully washed with running water for subsequent measurements of growth rate, dry weight, chlorophyll and carotenoid content, leaf area, and morphoanatomical analysis of the leaves.

The measurement of the length of the roots and shoots (epicotyl and hypocotyl) of young *H. impetiginosus* plants was carried out using a ruler graduated in millimeters. For the length of the hypocotyl, the measurement was taken from the base of the collar to the cotyledon, and for the epicotyl, from the cotyledon to the apex of the plant. Root length was measured from the base of the collar to the end of the root (mm). For dry weight, the aerial parts were sectioned from the roots, placed in paper bags, and taken to an air circulation oven for 30 min at 105°C, and for 24 h at 80°C, and weighed on an analytical scale [78]. Leaf area was determined using ImageJ software (version 1.54h) by taking photographs of the leaf blades of 10 seedlings from each treatment.

For the extraction and quantification of chlorophyll and carotenoid content in the leaves of *H. impetiginosus* seedlings, 100 mg of fresh leaves were used, with the veins removed. In a dark environment (lights off), the samples were macerated with calcium carbonate (CaCO_3_) and 80% acetone (10 mL) using a mortar and pestle. After maceration, the samples were centrifuged for 3 min at 200 × *g*, and the supernatant of each sample was analyzed for absorbances of 470 nm (carotenoids), 646 nm (chlorophyll *a*), 663 nm (chlorophyll *b*), and 710 nm (total chlorophyll) [107, 108]. The concentrations of chlorophyll *a* and *b*, total chlorophyll, and carotenoids were calculated according to the method proposed by [109].

#### 4.6.3 Morphoanatomical Changes

To analyze the morphoanatomical changes of the young stems and radicle, seedlings were collected after 15 days of initiating the germination test. For the leaves, they were analyzed after 2 months (60 days) of the growth test. All parts of the plant analyzed underwent the fixation process in FAA 50 (formaldehyde, glacial acetic acid, and 50% ethanol, v/v) for 48 h and were subsequently stored in 70% ethyl alcohol, following the method proposed by [110].

The anatomical sections were performed freehand, set up at 4 mm below the collar for the radicle, and 4 mm below the cotyledons for the young stem, as the standard for starting points for the sections. Expanded leaves were selected, and was used the second pair of leaves from the young plant. For anatomical analysis, sections of five radicles and five young stems per repetition were used. For leaf, five pairs of leaves per treatment.

Staining was performed using fuchsin and astra blue [111]. Samples were placed in glycerinated gelatin between the slide and coverslip and examined using an optical microscope (objectives: 10×, 40×, and 100×) coupled to a computer, and analyzed using the Motic software version 3.0. Several variables were observed in cross‐sections, such as the thickness of the epidermis, cortex, and vascular cylinder (young stem and radicle), the thickness of the palisade parenchyma, spongy parenchyma, abaxial and adaxial epidermis (leaf). These measurements were carried out for a better understanding of how allelopathic compounds affected the morphology of the recipient species. Qualitative and quantitative analyses of tissue alteration and thickness were carried out.

#### 4.6.4 Statistical Analysis

To assess the normality of the data, D'Agostino–Pearson, Shapiro–Wilk, and Kolmogorov–Smirnov tests were performed. Normal data were subjected to analysis of variance (the ANOVA test), and the measurements were compared using the Tukey test at 5% probability (*p* < 0.05). The data that did not meet normality parameters were subjected to the Kruskal–Wallis test. All data were analyzed using GraphPad Prism 6.0 software [112].

## Author Contributions

**Bruno Melo de Alcântara**: conceptualization, methodology, formal analysis, investigation, writing – original draft preparation. **Paulo Henrique Calixto Santana**: methodology, statistical analysis. **Felipe Rufino dos Santos**: methodology, statistical analysis. **José Weverton Almeida‐Bezerra**: writing – review methodology, statistical analysis. **José Galberto Martins da Costa**: writing – review methodology, statistical analysis. **Delmira da Costa Silva**: methodology, writing – review and editing. **Cláudia Maria Furlan**: methodology, writing – review and editing. **Maria Arlene Pessoa da Silva**: methodology, writing – review and editing, supervision.

## Conflicts of Interest

The authors declare no conflicts of interest.

## Data Availability

The data that support the findings of this study are available from the corresponding author upon reasonable request.
